# Improved Utilization of Nitrate Nitrogen Through Within-Leaf Nitrogen Allocation Trade-Offs in *Leymus chinensis*

**DOI:** 10.3389/fpls.2022.870681

**Published:** 2022-04-28

**Authors:** Xiaowei Wei, Yuheng Yang, Jialiang Yao, Jiayu Han, Ming Yan, Jinwei Zhang, Yujie Shi, Junfeng Wang, Chunsheng Mu

**Affiliations:** ^1^Key Laboratory of Vegetation Ecology of the Ministry of Education, Jilin Songnen Grassland Ecosystem National Observation and Research Station, Institute of Grassland Science, Northeast Normal University, Changchun, China; ^2^Key Laboratory for Plant Resources Science and Green Production, Jilin Normal University, Siping, China

**Keywords:** leaf N allocation, nitrate, ammonium, photosynthetic nitrogen-use efficiency, cell wall, *Leymus chinensis*

## Abstract

The Sharply increasing atmospheric nitrogen (N) deposition may substantially impact the N availability and photosynthetic capacity of terrestrial plants. Determining the trade-off relationship between within-leaf N sources and allocation is therefore critical for understanding the photosynthetic response to nitrogen deposition in grassland ecosystems. We conducted field experiments to examine the effects of inorganic nitrogen addition (sole NH_4_^+^, sole NO_3_^–^ and mixed NH_4_^+^/NO_3_^–^: 50%/50%) on N assimilation and allocation by *Leymus chinensis*. The leaf N allocated to the photosynthetic apparatus (N_PSN_) and chlorophyll content per unit area (Chl_area_) were significantly positively correlated with the photosynthetic N-use efficiency (PNUE). The sole NO_3_^–^ treatment significantly increased the plant leaf PNUE and biomass by increasing the photosynthetic N allocation and Chl_area_. Under the NO_3_ treatment, *L. chinensis* plants devoted more N to their bioenergetics and light-harvesting systems to increase electron transfer. Plants reduced the cell wall N allocation or increased their soluble protein concentrations to balance growth and defense under the NO_3_ treatment. In the sole NH_4_^+^ treatment, however, plants decreased their N allocation to photosynthetic components, but increased their N allocation to the cell wall and elsewhere. Our findings demonstrated that within-leaf N allocation optimization is a key adaptive mechanism by which plants maximize their PNUE and biomass under predicted future global changes.

## Introduction

Nitrogen (N) plays a vital role in ecosystems. This mineral element is required for plant growth and is typically absorbed as ammonium (NH_4_^+^) or nitrate (NO_3_^–^). Ammonium N (NH_4_^+^), and nitrate (NO_3_^–^) are also the main forms of N loading associated with atmospheric deposition (Galloway et al., 2008; Stevens, 2019; Liang et al., 2020). The N-use strategies of plant species of different functional types vary, and different plants thus respond differently to N additions (Xia and Wan, 2008) as the grasses acquire N from the soil and adopt more flexible strategies for different soil N sources to meet their high N demand (Callow, 1999). Generally, larger plant growth responses to NH_4_^+^-N than NO_3_^–^-N addition have been found in terrestrial plants, but not in shrubs or grasses (Yan et al., 2019; Liang et al., 2020). However, the differences in the N form uptaken by different species (Marschner and Marschner, 2012; Grassein et al., 2015) are likely to reflect differences in the N uptake and N use efficiency of the species (Lu et al., 2021). The availability of co-provisional NO_3_^–^ affects the accumulation and assimilation of NH_4_^+^ in roots and leaves (Prinsi and Espen, 2018). Uptake of NH_4_^+^ and NO_3_^–^ is mediated by low and high affinity systems in higher plants (Haynes and Goh, 1978; Forde, 2000; Howitt and Udvardi, 2000). The uptake and utilization of NH_4_^+^-N and NO_3_^–^-N by plants is critical for agricultural production and ecosystem stability (Tho et al., 2017; Luo et al., 2021).

The metabolism of carbon and N are interactively coupled across scales, from the leaf scale to the whole plant scale. Thus, changes in the availability of N at one of these scales are likely to affect the metabolic system at other scales (Liang et al., 2020). The assimilation NH_4_^+^ and NO_3_^–^ affects several biochemical and molecular mechanisms, thus altering various specific physiological processes throughout the plant development process (Liu and von Wirén, 2017). The majority of species are sensitive to excess NH_4_^+^ because less energy is required to uptake this form, but at high concentrations, this molecule might trigger numerous metabolic disorders (Britto and Kronzucker, 2002; Hessini et al., 2013). Generally, plants exposed to excess NH_4_^+^ and NO_3_^–^ display reduced growth, increased N metabolism-related enzymes, and modified photosynthetic physiological characteristics (Guo et al., 2008; Mu and Chen, 2021). Nitrate reductase (NR), nitrite reductase (NiR), Glutamine synthetase (GS) I, and GSII activities and the transcriptional levels of the corresponding genes in wheat seedlings are significantly reduced by N deficiency (Balotf et al., 2016). In general, the activity of N metabolism enzymes is significantly related to the synthesis of photosynthesis (Marschner, 2012). The results of a meta-analysis showed that the effects of N deposition on 14 photosynthesis-related traits and affecting moderators and the associated plant trait responses depended on biological, experimental, and environmental moderators (Liang et al., 2020). Moderators that affect the responses of photosynthetic N metabolism have less been simultaneously considered in previous studies.

N is absorbed by plants and distributed in plant leaves in different forms, such as soluble components (e.g., nitrates, amino acids, and proteins) and insoluble components (e.g., cell walls, membranes, and other structures; Feng et al., 2009; Liu et al., 2018). Approximately half of the total leaf N is used for photosynthesis and is allocated to three main systems: the carboxylation, bioenergetics, and light harvesting systems (Hikosaka and Terashima, 1995; Takashima et al., 2004). Small changes in photosynthetic N can affect the carboxylation efficiency and photosynthetic N use efficiency (PNUE) of plants (Feng et al., 2009; Onoda et al., 2017). Cell walls are a major N sink in leaves and are used for plant defense (Evans and Poorter, 2001; Feng et al., 2009). Mass and thickness of cell wall changed in response to sink–source perturbation, which caused decreases in gm and photosynthesis in soybean and French bean (Sugiura et al., 2020). Many studies have focused on the leaf N allocation trade-offs among different leaf components (Takashima et al., 2004; Feng et al., 2009; Onoda et al., 2017). For example, invasive species allocate more leaf N to their carboxylation and bioenergetics systems than native species, leading to invasive plants having higher A_*n*_, PNUE, and respiration efficiencies (Feng, 2008, Feng et al., 2009). The invasive species generally had lower LMA than natives, allocate more N to soluble protein, amino acids, and nucleic acids and less N to cell wall protein, aligning them closer to the “high-return” end of the leaf economics spectrum (Funk et al., 2013). Maize plants tend to invest relatively more N into bioenergetics to sustain electron transport under low-N-stress conditions (Mu et al., 2016). This suggests that plants were able to optimally allocate their nutrients to achieve an adaptive “functional balance.” Storage N is used for coordinating leaf expansion and photosynthetic capacity in winter oilseed rape (*Brassica napus* L.) from emergence to senescence, thereby promoting leaf growth and biomass (Liu et al., 2018). The mechanisms by which NH_4_^+^-N and NO_3_^–^-N are allocated and utilized in the photosynthetic carbon assimilation process have rarely been studied.

Grasslands play an important role in coping with global change (Liu et al., 2019; Shi et al., 2021). *Leymus chinensis* is a perennial rhizomatous grass that is often considered the foundational and dominant species in the eastern Eurasian steppe regions (Zhu, 2004). Additionally, in these regions, the N availability in the soils is often limited. Although N preferences have been studied in relatively few grassland species, these responses of grassland plants to N availability and relative preferences for NH_4_^+^ and NO_3_^–^ are important in structuring natural grassland communities (Cui et al., 2017), but have also become of recent interest in managed grasslands. Adding a small amount of NH_4_^+^-N to NO_3_^–^-N can significantly affect the photosynthesis, growth, and biomass accumulation of *L. chinensis* (Zhang et al., 2018). In addition, other studies have shown that NH_4_^+^-N is more suitable for *L. chinensis* growth than NO_3_^–^-N or glycine (Li et al., 2018). The results of previous studies on the effects of NH_4_^+^-N to NO_3_^–^-N on the growth and biomass accumulation of *L. chinensis* have extensively varied.

This study aimed to clarify the trade-offs of within-leaf N allocation to the upregulation of photosynthesis responding to the varying N supply conditions. To date, studies on the effects of N forms have mainly focused on plant preference and root growth (Gansel et al., 2001; Leghari et al., 2016; Cui et al., 2017; Kumar et al., 2020), whereas few have reported its effects on N assimilation and absorption and within-leaf N allocation. In the present study, the effects of different N forms (sole NH_4_^+^, sole NO_3_^–^ and mixed NH_4_^+^/NO_3_^–^: 50%/50%) supply on leaf N assimilation and within-leaf N allocation were examined under field conditions to elucidate the physiological mechanism of NO_3_^–^-N assimilation and leaf N allocation in *L. chinensis* leaves, and to enrich the theory of N absorption in *L. chinensis* leaves.

## Materials and Methods

### Plant Materials and Growth Conditions

The field experiment was carried out at the Jilin Songnen Grassland Ecosystem National Observation and Research Station in Jilin Province, Northeast Normal University, China (44°34’N, 123°31’E). The experimental site was located in the semi-arid, semi-humid, and temperate continental monsoonal climate zone. The study area was characterized by hot and rainy summers and cold and dry winters. The soil properties in 0–20 cm soil layer were as follows: pH 8.75; EC, 79.16 μs cm^–1^; total N, 1.04 g kg^–1^; total phosphorous (P); 68 g kg^–1^; organic Carbon (C), 6.43 g kg^–1^; NH_4_^+^-N 1.24 mg kg^–1^; NO_3_^–^-N 1.91 mg kg^–1^. The mean temperature ranges from 4.6 to 6.5°C. The annual mean precipitation ranges from 280 to 620 mm, with the majority of rainfall falling between June and September, and the mean annual rainfall ranging from 1,200 to 1,300 mm (Guo et al., 2020; Shi et al., 2021). The pot experiment was conducted according to a complete randomized block design with six replicates, with the plastic pots (15 cm in diameter and 25 cm in depth) filled with chestnut soil (3.5 kg soil pot ^–1^).

*Leymus chinensis* (Trin.) Tzvel. (C_3_ perennial rhizomatous grass) was widely distributed in northern China, eastern Mongolia, Transbaikalia, and Russia. It has good ecological adaptability and tolerance to drought, saline-alkali, and low temperature environment. Thus, it often forms *L. chinensis* steppes and meadows as a dominant species (Liu et al., 2019). On April 20, shoots of *L. chinensis* were transplanted into plastic pots, while shoots were collected from the eastern of Eurasia meadow steppe. Based on the investigation of the population density of natural *L. chinensis* grassland in the field experimental site during the green period (April 10- May 10), all species were planted with four individuals per pot in monoculture, and the plots were harvested on August 20. Additional N was applied at four different treatment levels: unfertilized treatment (N0), sole NH_4_^+^-N [as (NH_4_)_2_SO_4_] (NH_4_), sole NO_3_^–^-N [as Ca(NO_3_)_2_] (NO_3_), and mixture of both NH_4_^+^-N and NO_3_^–^-N in ratio of 1:1 (NH_4_NO_3_) for a total of 10 g N m^–2^. Two equal portions of each mixture was added into each pot (May 10 and June 6). In the previous research conducted in the north grassland, N deposition at 10 g N m^–2^ y^–1^ was the maximum amount (Zhang et al., 2017). The medium containing NH_4_^+^ as the only N source was buffered with CaCl_2_ (39.7 g m^–2^). In addition, the nitrification inhibitor dicyandiamide (DCD, 98.0%) was added to the NH_4_^+^ (10 mg m^–2^ y^–1^) and NH_4_NO_3_ treatment (5 mg m^–2^ y^–1^) to inhibit nitrification of NH_4_^+^. Other fertilizers (P, K, S) and micronutrients (Zn, B, Mn, Mo, Cu, and Fe) were applied for all treatments to ensure that plant growth was not limited by nutrients other than N. The plots were kept free of weeds, insects, and diseases during the growth season, and all mesocosms were exposed to natural precipitation events and less irrigation to ensure normal plant growth. The plots were harvested on August 20 during the post fruiting vegetation growth stage.

### Gas Exchange Measurements and Chlorophyll Fluorescence

From 24 to 30 July 2019, the leaf assimilation rate (A_*n*_, μmol m^–2^ s^–1^), stomatal conductance (g_*s*_, mmol m^–2^ s^–1^), and internal CO_2_ (C_*i*_, μmol mol^–1^) were measured using a CIRAS-3 portable photosynthesis system (PP Systems, United States) equipped with a CO_2_ concentration at 400 μmol mol^–1^ in the leaf chamber, at 500 μmol s^–1^ flow rate, and at 25°C. The photosynthetic photon flux density (PPFD) of the leaf chamber was set to 1,600 μmol m^–2^ s^–1^ (with 90% red light, 5% blue light, and 5% white light) and 65% relative humidity. For the rapid A/C_*i*_ response curve (Stinziano et al., 2017), the CO_2_ partial pressure was changed from 50 to 1,200 μmol mol^–1^. In each pot, the 2nd and 3rd leaf from the tip of the shoot were used for leaf gas exchange measurements and conducted between 8:00 a.m. and 16:00 a.m. (six replicates).

The maximum rate of Rubisco carboxylation (V_*cmax*_, μmol m^–2^ s^–1^) and maximum rate of electron transport (J_*max*_, μmol m^–2^ s^–1^) were calculated by the A/C_*i*_ curves data and fitted by using the models of von Caemmerer (2000) and Long and Bernacchi (2003). The details were calculated as follows:


$${\text{V}}_{\text{cmax}}=\frac{\left({\text{R}}_{\text{d}}+{\text{A}}_{\text{n}}\right)\,\left[{\text{C}}_{\text{i}}+{\text{K}}_{\text{C}}\,\left(1+\frac{\text{O}}{{\text{K}}_{0}}\right)\right]}{\left({\text{C}}_{\text{i}}-\Gamma^{*}\right)}$$

$${\text{J}}_{\text{max}}=\frac{4\,\left({\text{R}}_{\text{d}}+{\text{A}}_{\text{n}}\right)\,\left({\text{C}}_{\text{i}}+2\,\Gamma^{*}\right)}{\left({\text{C}}_{\text{i}}-\Gamma^{*}\right)}$$

where R_*d*_ is the mitochondrial respiration rate in the light (μmol m^–2^ s^–1^), K_*c*_ and K_*o*_ are Michaelis constants for carboxylation and oxygenation, O is the intercellular oxygen concentration close to 210 mmol mol^–1^, and Γ* is the CO_2_ compensation point in the absence of respiration (μmol mol^–1^), Additionally, K_*c*_, K_*o*_, and Γ* calculated by the temperature dependence function from Bernacchi et al. (2001, 2003).

The chlorophyll fluorescence was obtained in order to analyze PSII quantum efficiency of plants by using an IMAGING PAM M-series (Walz, Effeltrich, Germany), and dark period of the samples was dark for 30 min before measurements. The maximum quantum yield of PSII (Fv/Fm), the effective quantum yield of PSII (φPSII), non-photochemical quenching coefficient (NPQ), and electron transport rate (ETR, μmol e^–1^ s^–1^ m^–2^) were calculated according to Zhou et al. (2021).

### Biochemical Measurements

After the determination of the chlorophyll fluorescence parameters, the leaf area was determined with a portable leaf area meter (AM350, ADC Bio Scientific Ltd., Herts, United Kingdom). Two leaves per plant were collected, immediately frozen in liquid N, and stored at -80°C for biochemical analysis. Two additional leaves were halted enzyme activity at 105°C for 30 min of leaves and dried to a constant weight at 65°C. Then biomass was measured and analyzed for total N content (N_*m*_, mg g^–1^) with an Elementar Vario EL Cube (Elementar, Langenselbold, Germany). A leaf mass per unit leaf area (LMA, g m^–2^) and a leaf N content per unit leaf area (N_area_, g m^–2^) were calculated as N_area_ = N_*m*_ × LMA. Chlorophyll per leaf mass (Chl_*m*_, mg g^–1^) was quantified by 0.1 g leaf in the ethanol extract, and measured using a spectrophotometer (UVmini*-*1240, Shimadzu, Japan) at 645 nm and 663 nm (Wellburn, 1994). The chlorophyll content was calculated as follows:


$${\text{Chl}}_{\text{a}}=12.43\times \mathrm{A663}-2.62\times \mathrm{A645}$$

$${\text{Chl}}_{\text{b}}=22.62\times \mathrm{A645}-4.36\times \mathrm{A663}$$

$${\text{Chl}}_{\text{m}}={\text{Chl}}_{\text{a}}+{\text{Chl}}_{\text{b}}$$

Chlorophyll per leaf area (Chl_area_) was calculated as Chl_area_ = Chl_*m*_ × LMA.

To quantify nitrate N and ammonium N contents in leaves, 2.0 g of lyophilized samples were incubated with 10 ml distilled water, boiled for 1 h, and filtered to obtain the crude extract. Subsequently, the NO_3_^–^ concentration was measured by the salicylic acid chromogenic method of Cataldo et al. (1975), while NH_4_^+^ concentration was determined by the phenol-hypochlorite method of Felker (1977). Free amino acid was measured by ninhydrin colorimetric method (Hwang and Ederer, 1975).

Different forms of N were measured according to Takashima et al. (2004) and Onoda et al. (2017) with some modifications. The leaves were powdered with liquid N and homogenized in 2 ml of Na-phosphate buffer (pH 7.5, 100 mmol L^–1^), then washed in a centrifuge tube. This procedure was repeated three times. The homogenates were centrifuged at 12,000 g at 4°C for 10 min, and the supernatant was regarded as soluble protein. The pellet was washed with 1 ml of phosphate buffer containing 3% sodium dodecyl sulfate (SDS), followed by centrifugation (12,000 g, 5 min) after heating in 90°C water for 5 min. This procedure was repeated six times while the supernatants regarded as SDS-soluble protein were collected. The residue, regarded as cell wall protein, was washed with ethanol into the quantitative filter paper. The supernatant was precipitated with 10% trichloroacetic acid (TCA) by heating at 85°C for 5 min. The precipitate was filtered with quantitative filter paper and washed with ethanol. The three types of components of N on the quantitative filter paper were dried at 85°C, and then analyzed by the Elementar Vario EL Cube.

Nitrate reductase, NiR, GSI, and GSII of frozen leaves was determined by plant NR, NiR, GSI, and GSII activity *ELISA* kit (Shanghai Enzyme Biotechnology Co., Ltd., China) according to the manufacturer’s instructions.

### Calculation of N Allocation in the Photosynthetic Apparatus and Photosynthetic N-Use Efficiency

According to the LUNA model developed by Niinemets et al. (1997, 2011), leaf photosynthetic N is divided into three major parts: the fractions of the total leaf N allocated to carboxylation system (PN_*C*_, g g^–1^), electron transport components (PN_*B*_, g g^–1^), and light harvesting components (PN_*L*_, g g^–1^). The photosynthetic apparatus were calculated as follows:


PNC=Vc⁢max 6.25×Vcr×Narea

$${\text{PN}}_{\text{B}}=\frac{{\text{J}}_{\text{max}}}{8.06\times {\text{J}}_{\text{mc}}\times {\text{N}}_{\text{area}}}$$

$${\text{PN}}_{\text{L}}=\frac{{\text{C}}_{\text{C}}}{{\text{N}}_{\text{area}}\times {\text{C}}_{\text{B}}}$$

where 6.25 (g Rubisco g^–1^ N) was the coefficient of Rubisco conversion into N at 25°C (Douglas et al., 1984), V_*cr*_ was 20.78 (μmol CO_2_ g^–1^ Rubisco s^–1^) at 25°C (Niinemets and Tenhunen, 1997), 8.06 was the N conversion coefficient of cytochrome (Nolan and Smillie, 1977), J_*mc*_ was the maximum electron transport rate per unit cytochrome f s^–1^ (155.65 μmol e^–1^ μmol cytochrome f s^–1^) at 25°C (Niinemets and Tenhunen, 1997; Niinemets et al., 2011), Cc was leaf chlorophyll content (mmol g^–1^), and C_*B*_ was chlorophyll binding to light harvesting components (2.15 mmol g^–1^ N; Hikosaka and Terashima, 1995). The fractions of leaf N allocated to the thylakoid (PN_*B* + *L*_, g g^–1^) and the photosynthetic apparatus (PN_PSN_, g g^–1^) were the sum of PN_*B*_ and PN_*L*_, and the sum of PN_*C*_, PN_*B*_, and PN_*L*_, respectively. N content in carboxylation (N_*C*_, g m^–2^), bioenergetics (N_*B*_, g m^–2^), light-harvesting system (N_*L*_, g m^–2^), and all components of the photosynthetic apparatus (N_PSN_, g m^–2^) were calculated as the products of PN_*C*_, PN_*B*_, PN_*L*_, and PN_PSN_ with N_area_, respectively. The remaining leaf N was defined as other N. Photosynthetic N use efficiency (PNUE, μmol g N^–2^ s^–1^) was calculated by A_*n*_/N_area_ (Poorter and Evans, 1998).

### Statistical Analysis

All data were examined for a normal distribution (Kolmogorov-Smirnov test) and homogeneity of variance (Levene’ s test) and conducted using R version 4.0.4 (R Core Team, 2020). Analyses were performed using the “Tukey’ s HSD” function from “agricolae” package and differences were considered significant at *p* < 0.05. A linear correlation was performed using “perason” function from the “ggpmisc” package. The biplot were plotted using the package “ggplot2.”

## Results

### Leaf Physiological and Morphological Traits

**FIGURE 1:**
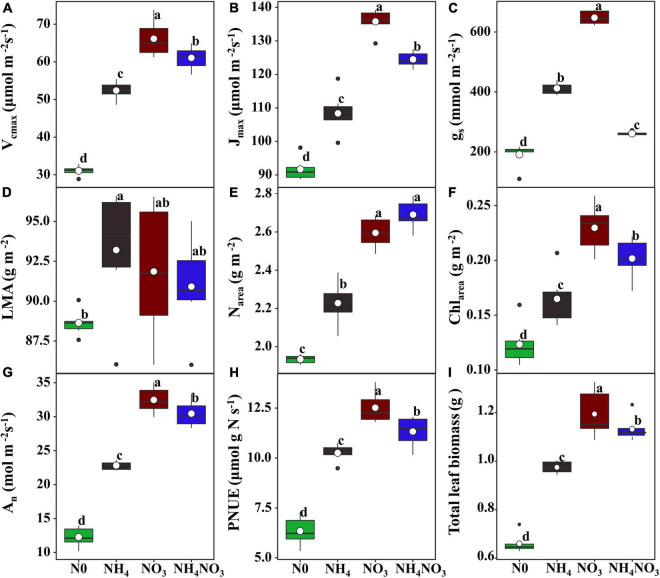
Effect of N (N) forms treatments on maximum carboxylation rate (V_*cmax*_) **(A)**, maximum photoelectron transfer rate (J_*max*_) **(B)**, stomatal conductance (g_*s*_) **(C)**, leaf mass area (LMA) **(D)**, area-based N content (N_area_) **(E)**, area-based chlorophyll content (Chl_area_) **(F)**, net CO_2_ assimilation rate (A_*n*_) **(G)**, photosynthetic N use efficiency (PNUE) **(H)**, and total leaf biomass **(I)** in *L. chinensis*. White dot is “Mean”; black dot is “Outlier”; horizontal is “Median”; the top of vertical line is “Max” and the bottom of vertical line is “Min.” Different lower-case letters indicate significant differences between the measuring dates under the unfertilized (N0) treatment and the fertilized (NH_4_, NO_3_, NH_4_NO_3_) treatment, respectively (*p* < 0.05) (*n* = 6).

The effects of N0, NH_4_, NO_3_, and NH_4_NO_3_ on V_*cmax*_, J_*max*_, and g_*s*_ were significant (*p* < 0.05) (Figure 1). The V_*cmax*_, J_*max*_, and g_*s*_ values of the NO_3_ treatment were significantly higher than those of the N0, NH_4_, and NH_4_NO3 treatments (*p* < 0.05) (Figures 1A–C). The leaf mass per area (LMA) measured under the NH_4_ treatment was significantly higher than under the N0 treatment, but no significant difference was found between NO_3_ and NH_4_NO_3_ (Figure 1D). The N0, NH_4_, NO_3_, and NH_4_NO_3_ treatments had significant effects (*p* < 0.05) on N_area_, Chl_area_, A_*n*_, and PNUE (Figures 1E–H). The N_area_ measured under the NO_3_ treatment was significantly higher than that under the N0 and NH_4_ treatments (*p* < 0.05), but no significant difference was found between the NO_3_ and NH_4_NO_3_ treatments (Figure 1E). The Chl_area_, A_*n*_, PNUE, and total leaf biomass measured under the NO_3_ treatment were significantly higher than those under the N0, NH_4_, and NH_4_NO_3_ treatments (*p* < 0.05) (Figures 1F–I).

### Leaf N Assimilation Enzyme Activity

**FIGURE 2:**
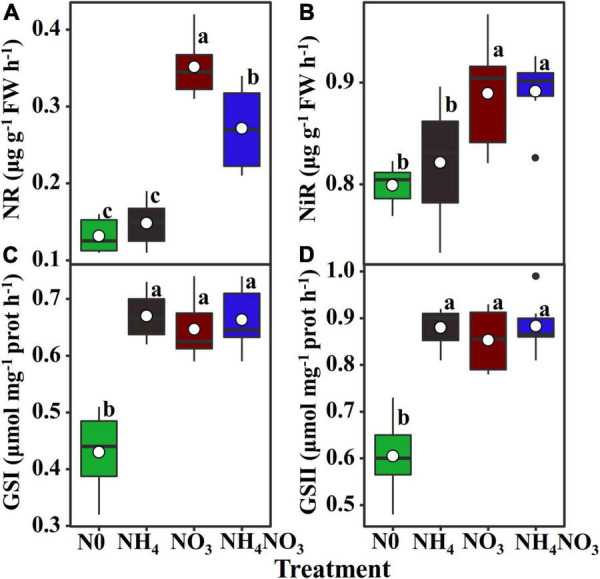
Effect of N forms treatments on the changes in activities of nitrate reductase, nitrite reductase, and glutamine synthetase isoforms of leaves in *L. chinensis*. **(A)** Nitrate reductase (NR; EC. 1.6.6.1/2), **(B)** nitrite reductiase (NiR; EC. 1.7.2.1), **(C)** glutamine synthetase (GS) I, and **(D)** GS II. White dot is “Mean”; black dot is “Outlier”; horizontal is “Median”; the top of vertical line is “Max” and the bottom of vertical line is “Min.” The changes in activities of enzyme under N0, NH_4_, NO_3_, and NH_4_NO_3_ treatments. Different lower-case letters indicate significant differences (*p* < 0.05) (*n* = 6).

To evaluate whether the induction of PNUE in the NH_4_^+^ and NO_3_^–^ supply treatments was related to nitrate and ammonium accumulation or to the induction of NR, NiR, and GS activity, NR and NiR activities were stimulated in the NO_3_ treatment. Conversely, they were inhibited in the NH_4_ treatment (Figures 2A,B). In contrast, neither the GSI nor the GSII isoform activity was changed due to the effects of different N forms despite presenting higher values compared to the N0 treatment (Figures 2C,D).

### Leaf N Allocation to Other Soluble-N Components

**FIGURE 3:**
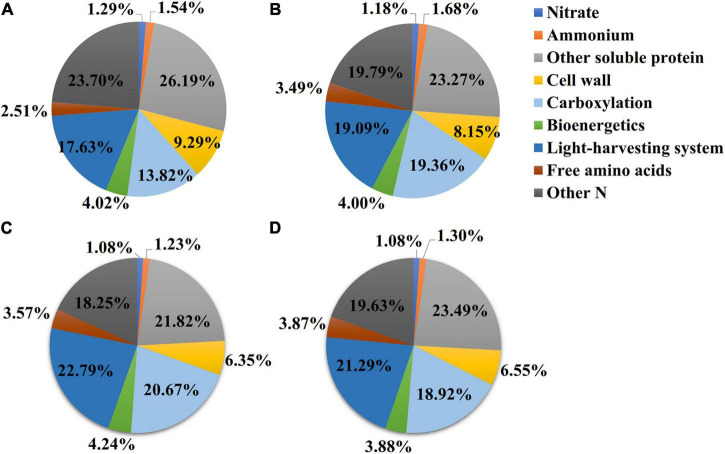
Effect of N forms treatments on the N allocation in leaves. The data of percentages are the content of N in the corresponding components accounting for total leaf N content in *L. chinensis*. N0-treated **(A)**, NH_4_-treated **(B)**, NO_3_-treated **(C)**, and NH_4_NO_3_-treated **(D)**. The size of pie chart indicates N content (*p* < 0.05) (*n* = 6).

The nitrate contents in the NO_3_- and NH_4_NO_3_-treated plants were higher than those measured in plants under the N0 and NH_4_ treatments (*p* < 0.05) (Table 1). However, in the NO_3_ treatment, the leaf nitrate content was very low, accounting for approximately 0.87% of the total leaf N (Figure 3C). The ammonium content measured under the NH_4_ treatment was higher than those measured under the treatments with other N forms (*p* < 0.05) (Table 1), accounting for approximately 1.36% of the total leaf N (Figure 3B). Compared with the NH_4_ treatment, the content of free amino acids was 21.07 and 31.44% higher under the NO_3_ treatment and NH_4_NO_3_ treatment. The amount of N measured in other soluble protein was 10.88 and 19.62% higher under the NO_3_ and NH_4_NO_3_ treatments than under the NH_4_ treatment (*p* < 0.05) (Table 1).

**TABLE 1:** Effect of nitrotgen (N) forms treatments on the content of N compounds in *L. chinensis*.

Parameters (mg m^–2^)	N forms treatment			
	
	N0	NH_4_	NO_3_	NH_4_NO_3_
Nitrate	23.38 ± 0.33 c	25.53 ± 0.49 b	27.63 ± 0.38 a	27.72 ± 0.34 a
Ammonium	27.87 ± 0.66 c	36.27 ± 0.25 a	31.42 ± 0.81 b	33.30 ± 0.78 b
Free amino acids	45.40 ± 2.04 d	75.31 ± 2.24 c	91.18 ± 1.70 b	98.99 ± 1.43 a
Other soluble protein	473.66 ± 6.61 b	502.66 ± 17.76 b	557.35 ± 15.47 a	601.33 ± 27.29 a
Cell wall	168.04 ± 1.71 ab	175.96 ± 0.90 a	162.05 ± 3.93 b	167.56 ± 4.17 ab
Carboxylation	249.86 ± 4.21 d	418.06 ± 7.71 c	527.81 ± 15.87 a	484.15 ± 8.57 b
Bioenergetics	72.75 ± 1.01 d	86.37 ± 2.06 c	108.23 ± 1.18 a	99.22 ± 0.74 b
Light-harvesting system	318.74 ± 18.21 c	412.23 ± 25.64 b	582.08 ± 21.91a	544.80 ± 31.87a
Other N	428.26 ± 8.38b	427.35 ± 18.53 b	466.18 ± 14.57 ab	502.34 ± 26.67 a
Total N (g kg^–1^)	21.82 ± 0.05 d	23.90 ± 0.23 c	28.28 ± 0.29 b	29.60 ± 0.11 a

*Data were reported as the arithmetic mean ± 1 standard error (n = 6). Numbers followed by different lower-case letters indicate significant differences, according to Tukey’s test (p < 0.05).*

### Leaf N Allocation to Structure-N Components

**FIGURE 4:**
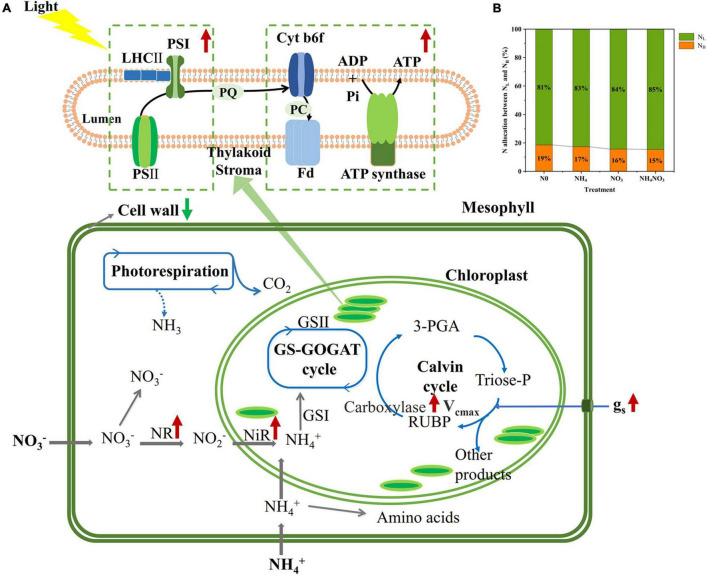
Effect of N forms treatments on the change of N contents in photosynthetic apparatus of leaves in *L. chinensis*. **(A)** The percentage together with indicate the increase (red arrows) and the reduction (green arrows) of N in different photosynthetic apparatus under NO_3_ compared to N0, NH_4_, and NH_4_NO_3_ treatments. **(B)** The allocation of N between PN_*B*_ and PN_*L*_ within the thylakoid lumen under N0, NH_4_, NO_3_, and NH_4_NO_3_ treatments.

**FIGURE 5:**
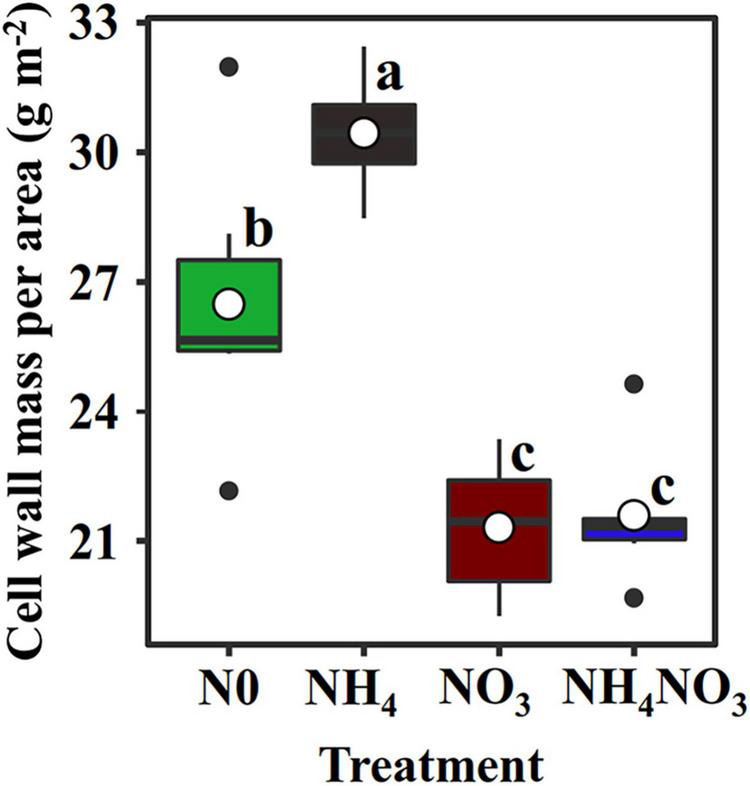
Effect of N forms treatments on cell wall mass per area in *L. chinensis*. White dot is “Mean”; black dot is “Outlier”; horizontal is “Median”; the top of vertical line is “Max” and the bottom of vertical line is “Min.” Different lower-case letters indicate significant differences under N0, NH_4_, NO_3_, and NH_4_NO_3_ treatments (*p* < 0.05) (*n* = 6).

The N_*C*_ (carboxylation) and N_*B*_ (bioenergetics) values expressed per unit leaf area were significantly higher under the NO_3_ treatments than under the N0, NH_4_, or NH_4_NO_3_ treatments (*p* < 0.05) (Table 1 and Figure 3). No significant difference was found in N_*L*_ (light-harvesting system) between the NO_3_ and NH_4_NO_3_ treatments, but N_*L*_ was significantly higher in these treatments than in the N0 and NH_4_ treatments (*p* < 0.05) (Table 1). Compared to the N0, NH_4_, and NH_4_NO_3_ treatments, N_*B*_/N_*B* + *L*_ decreased under the NO_3_ and NH_4_NO_3_ treatments, while N_*L*_/N_*B* + *L*_ increased (*p* < 0.05) (Figure 4B). The leaf cell wall N content (N_*cw*_) was 7.91% lower in the NO_3_ treatment than in the NH_4_ treatment (Table 1), while the cell wall per area was higher in the NH_4_ treatment (*p* < 0.05) (Figure 5).

### Within-Leaf N Allocation Estimate

**FIGURE 6:**
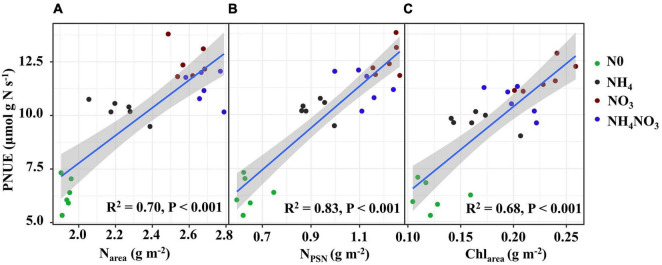
Relationships of photosynthetic N use efficiency (PNUE) with area-based N content (N_area_) **(A)**, photosynthetic N (N_PSN_) **(B)** and area-based chlorophyll content (Chl_area_) **(C)** in *L. chinensis*. The color of green, black, red, and blue correspond to the N0, NH_4_, NO_3_, and NH_4_NO_3_ treatments. Relationships between variables were assessed using linear regression analysis.

The effects of different available N forms on the allocation of leaf N to different N components are shown in Figure 3. Relative to the NH_4_ and NH_4_NO_3_ treatments, the NO_3_ treatment significantly increased the percentages of N allocated to carboxylation (1.31 and 1.75%, respectively), bioenergetics (0.24 and 0.36%), and light-harvesting system (3.7 and 1.5%) proteins. Unexpectedly, the amounts of N allocated to the nitrate and other soluble protein N components were elevated under NO_3_ treatment. The percentage of N in free amino acid was 1.06 and 0.08% higher under NO_3_ treatment than NH_4_ and NH_4_NO_3_ treatments. Assessing the other N proportions, under the NO_3_ treatment, the N proportions were 5.45, 1.54, and 1.38% lower than those measured under the N0, NH_4_, and NH_4_NO_3_ treatments, respectively. The percentage of N allocated to cell walls exhibited a similar trend as the cell wall biomass under the different N forms. In summary, the correlation analyses revealed highly active relationships between N_area_ and PNUE and between N_PSN_ and PNUE (Figures 6A,B).

### PSII Quantum Efficiencies

**FIGURE 7:**
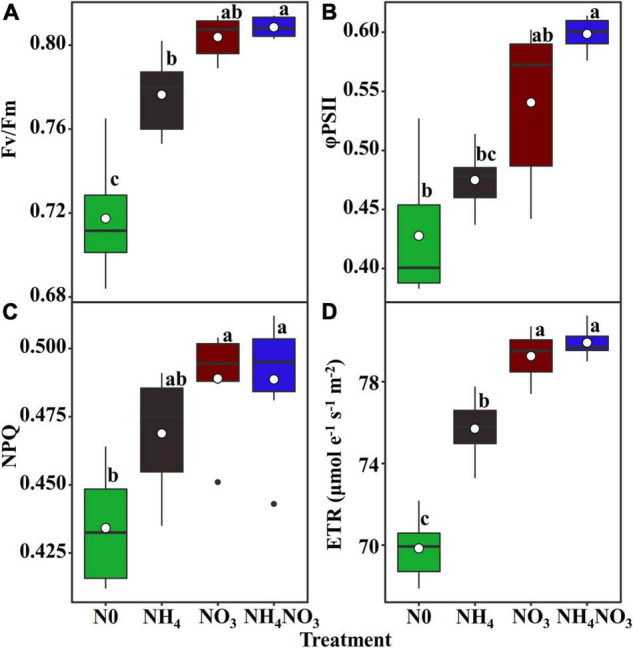
Effect of N forms treatments on the maximum quantum yield of PSII (Fv/Fm) **(A)**, the effective quantum yield of PSII (φPSII) **(B)**, non-photochemical quenching coefficient (NPQ) **(C)**, and electron transport rate (ETR, μmol e^–1^ s^–1^ m^–2^) **(D)** in *L. chinensis*. White dot is “Mean”; black dot is “Outlier”; horizontal is “Median”; the top of vertical line is “Max” and the bottom of vertical line is “Min.” Different lower-case letters indicate significant differences under N0, NH_4_, NO_3_, and NH_4_NO_3_ treatments (*p* < 0.05) (*n* = 6).

Since *L. chinensis* plants exhibited an advantage characterized by allocating N to photosynthetic components in leaves under the NO_3_ treatment, we investigated whether nitrate and ammonium affect the PSII quantum efficiencies. Positive and highly significant linear relationships between PNUE and Chl_area_ were observed in *L. chinensis* (Figure 6C). The Fv/Fm, φPSII, non-photochemical quenching (NPQ), and electron transfer rate (ETR) were significantly higher under the NO_3_ and NH_4_NO_3_ treatments than under the NH_4_ and N0 treatments (*p* < 0.05) (Figure 7).

## Discussion

In this study, a set of experimental observations was conducted on the photosynthetic responses of *L. chinensis* (a C_3_ plant) to varying N nutrient sources to capture leaf economics spectrum response mechanism. For a better understanding of absorption and utilization of nitrate N, observations ranged from plants’ morphological features, trough overall photosynthesis, and within-leaf N allocation, up to photosynthetic component N and nutrient concentration in plants tissues. During the growing season, NH_4_^+^ and NO_3_^–^ strongly affected each of investigated aspects of plant functioning and development.

As is well documented, N is an essential nutrient in plant growth and development, and its form can affect leaf growth (Cui et al., 2017). Leaf morphological adjustments are generally recognized to be more striking than leaf biochemical characteristics in determining leaf photosynthesis adaptations to the environment (Niinemets et al., 2011; Onoda et al., 2017). N promotes leaf area growth and helps leaves absorb light energy, thereby contributing to the maintenance of A_*n*_ and PNUE (Poorter and Evans, 1998; Onoda et al., 2017). The NO_3_-treated plants showed higher g_*s*_ values than the plants exposed to other treatments. As expected, the increased g_*s*_ affected CO_2_ assimilation and the higher V_*cmax*_ values suggest that biochemical restrictions should have also been reduced. According to Guo et al. (2003), nitrate is a well-known anionic transporter involved in the stomatal opening mechanism. This result also illustrates that the NO_3_-treated plants had higher g_*s*_ values than the NH_4_-treated plants. In the present study, *L. chinensis*, as a group, had no significant LMA with higher A_*n*_, Chl_area_, and N_area_ under NO_3_ treatment compared to the N0, NH_4_, and NH_4_NO_3_ treatments, resulting in the PNUE improving by 22.02 and 10.51%, respectively. In support of this idea, in *L. chinensis*, PNUE was positively correlated with N_area_, N_PSN_, and Chl_area_. V_*cmax*_ is a proxy for the enzymatic activity of Rubisco during the photosynthetic carbon-fixation reactions (Farquhar et al., 1980; Sharkey, 2016; Zhuang et al., 2021). The inorganic N sources significantly increased the V_*cmax*_ and J_*max*_ of *L. chinensis*. Variations in V_*cmax*_ can be explained by changes in LMA, N_area_, or the proportion of N allocated to the carboxylation system (Yin et al., 2019; Zhuang et al., 2021). These findings indicated that the NO3- supply is closely related to the normal growth of *L. chinensis* leaves.

Nitrate reductase and NiR participate in the process of reducing NO_3_^–^ to NH_4_^+^ in coupled regulation (Kovács et al., 2015). In our study, the NO_3_ treatment strongly stimulated the NR and NiR activities. This finding is consistent with previous studies reporting that NR activity is mainly affected by the concentration of NO_3_^–^ (Balotf et al., 2016; Wen et al., 2019). When NO_3_^–^ is converted to other forms of N, the availability of NO_3_^–^ decreases, but the N in the soil was continuously transferred to the leaves, which led to an increase in the NO_3_^–^ content and NR and NiR activities (Britto and Kronzucker, 2002; Marschner, 2012). In higher plants, GSI and GSII assimilate NH_4_^+^ into amino acids for plant absorption and utilization in leaves (Bloom, 2015). Interestingly, although the concentration of NH_4_^+^ is closely related to GSI and GSII enzyme activities (Forde and Clarkson, 1999), GSI and GSII enzyme activities have no significant difference under N supply treatments, as has been previously reported for rice plants (Alencar et al., 2019; Sugiura et al., 2020). The results of this study reveal the relationships between the NO_3_^–^ and NH_4_^+^ supply with assimilation enzyme activity. According to our results, the enzyme activity of N isozyme significantly increased under NO_3_^–^ treatment.

Intra-leaf N allocation should reflect trade-offs in the economic spectrum of leaves, with faster-growing species allocating more N to metabolism at the expense of structure (Funk et al., 2013). Thus, we hypothesized that *L. chinensis* under NO_3_- treatment, which are generally located on the “high-return” of the leaf economics spectrum, would have higher A_*n*_, N_area_, and PNUE relative to other treatments. Therefore, it has greater allocation to leaf N pools associated with photosynthesis and growth. Species with greater N investments in photosynthetic proteins generally show higher PNUE in many natural ecosystems (Feng, 2008, Feng et al., 2009; Shi et al., 2019). Based on our original assumption of “high-return,” we must assess the changes in the leaf N allocation process. In ecological models, N investments in the photosynthetic apparatus remain an important PNUE determinant (Feng et al., 2009; Liu et al., 2018). Photosynthesis is closely related to the leaf N content, which can be directly reflected by Calvin cycle proteins. Approximately three-quarters of leaf N is distributed to the photosynthetic apparatus (Dubreuil et al., 2017; Bahar et al., 2018; Zhang et al., 2020). In this study, *L. chinensis* allocated 47.7% of leaf N to the photosynthetic apparatus, and this was in accordance with previously reported results for rice plants (Zhong et al., 2019) and invade plants (Feng, 2008). Furthermore, we found that the amount of leaf N allocated to the photosynthetic apparatus was significantly positively correlated with PNUE (*R*^2^ = 0.83, *p* < 0.001). *L. chinensis* leaves have lower cell wall protein with higher amino acid content under NO_3_-treated plants, consistent with allocation to growth at the expense of structure. However, our hypothesis that *L. chinensis* leaves would allocate more resources to carbon assimilation and growth at the expense of structure was only partially supported under NO_3_-treated plants. *L. chinensis* also had higher amounts of total N and membrane-bound protein.

Nitrate treatment caused a relative increase in content of other soluble protein N and carboxylation N and the percentage (42.49%) of total soluble protein-N in total leaf N, similar to the result of Makino et al. (2003), who reported that 25–45% of leaf N was allocated to soluble proteins. Soluble proteins and free amino acids are two of the most abundant N sources, and they store N in leaves (Liu et al., 2018). Among soluble proteins, Rubisco is a key enzyme involved in C_3_ photosynthesis (composing up to 50% of the leaf soluble protein and 25% of the leaf N; Lin et al., 2014). In the present study, the high photosynthetic N (N_PSN_) and low cell wall N (N_*CW*_) measured under the NO_3_ treatment were presumably associated with a decrease in the cell wall biomass fraction (Table 1 and Figure 5). Our finding that NO_3_- treatment and other treatments have significant differences in the allocation of N to soluble protein, consistent with previously published results that faster grow species allocated more N to soluble protein at the expense of cell-wall protein (Feng et al., 2009; Landi and Esposito, 2017). Previous studies have highlighted that cell walls are a part of the plant apoplast, which is also an important N sink that can defend plants against stress (Feng et al., 2009; Shang et al., 2019). These results suggest that the allocation of N to cell walls was decreased under NO_3_ conditions, thus possibly contributing to the increased absorption and utilization of N and the maintenance of photosynthesis in mesophyll cells to the greatest extent possible. The N investment strategy regarding these N components was changed under NO_3_ conditions, suggesting that these components are essential for ensuring adaptations of normal growth and physiological activities to inorganic N.

The NO_3_^–^-N used in our field experiment resulted in relatively even allocation of N to photosynthetic apparatus (e.g., carboxylation, bioenergetics, and light-harvesting components) and carbon assimilation (e.g., soluble protein, free amino acids) functions. Our data matched the theoretical estimates modeled from photosynthetic data, indicating that C_3_ plants invest about 24% leaf N to thylakoids and allocate 75% of thylakoids N to light harvesting proteins and 25% in bioenergetics (Poorter and Evans, 1998; Makino et al., 2003; Zhong et al., 2019; Mu and Chen, 2021). There are two types of thylakoid N, namely, one related to the bioenergetics system, such as the electron transport chain and photosynthetic phosphorylation, and another involved in the light-harvesting component (Mu et al., 2016). The absolute N content was devoted to biogenetics and light harvesting under the NO_3_ treatment. Relatively more N from the thylakoid was allocated to bioenergetics under the different N treatments. *L. chinensis* leaves had higher A_*n*_ and V_*max*_ compared under NO_3_-treated with other treatments. This suggests that Rubisco content or activity may have been higher in *L. chinensis* leaves. Our carboxylation fraction includes Rubisco, but Rubisco was not directly measured in this study. This proved that a leaf prioritization process occurred for the stabilization of the light harvesting and electron transfer systems under the NO_3_ treatment and thus the maximization of the PSII quantum yield (Antal et al., 2010; Wang F. et al., 2019; Wang P. et al., 2019). This conclusion is supported by the finding that the Fv/Fm, φPSII, and ETR values were significantly different under the NO_3_ treatment. Similarly, the higher NPQ measured under the NO_3_ treatment should have helped dissipate excess electrons. The NO_3_ treatment coincided with a higher leaf N concentration, and more N allocated to carboxylation compared to the other N treatments. It is likely that the relatively higher N in the bioenergetics and light-harvesting systems were well matched with the higher carboxylation capacity, promoting an increase in the photosynthetic rate and PNUE.

Our study examined within-leaf N partitioning in *L. chinensis* of the grassland dominant species in inorganic N absorption. *L. chinensis* leaves may succeed by allocating N to growth at the expense of higher leaf level carbon assimilation under NO_3_^–^ treatment. Furthermore, the leaf N assimilate enzyme activity and within-leaf N allocation were observed to exhibit different trends in response to the NO_3_ treatment compared to the other treatments (Figure 4), suggesting that the trade-off between N assimilation and N allocation was specific and dependent on the prioritization of N forms for absorption in the plants. The proportion of the cell wall N allocation and other N to growth decreased under the NO_3_ treatment. Under the NO_3_ treatment, the proportions of N allocated to soluble proteins and the photosynthetic system increased, whereas the amount of N allocated to the cell wall was reduced, characterizing a trade-off between growth and defense in *L. chinensis*. In this vein, we analyzed whether NO_3_^–^ supply was able to induce PNUE improvement in leaves to establish if these changes could have contributed to promoted plant growth. The enzyme activity of N isozyme was significantly increased under NO_3_^–^ treatment. However, further accurate studies employing additional and more systematic approach are needed to definite the different NO_3_^–^ concentrations effected the leaf N allocation.

## Conclusion

Our results evidence that NO_3_^–^ supply causes changes in some important photosynthetic processes in *L. chinesis* leaves. NO_3_^–^ induced increased in the NR and NiR enzyme activity which could have improved the process of reducing NO_3_^–^ to NH_4_^+^. N allocation was optimized within *L. chinensis* leaves, thus exhibiting an evolutional adaptation mechanism regarding the utilization of N for photosynthesis, thus increasing the PNUE and biomass during the growing season under NO_3_ environment. Under the NO_3_ treatment, *L. chinensis* plants tended to devote relatively more N to bioenergetics and the light-harvesting system to increase their ETR. Moreover, Chl_area_ and NPQ were increased to reduce the damage caused by excess electron production. Within-leaf N allocation should reflect trade-offs in *L. chinensis* on the leaf economics spectrum with allocating more N to metabolic processes at the expense of structure. Taken together, the results of our study provide a comprehensive picture of the effects of nitrate N on within-leaf N assimilation and allocation and can help researchers obtain a better understanding of the mechanisms by which *L. chinensis* in meadow grasslands absorb and utilize NO_3_^–^-N under the context of increasing N deposition.

## Data Availability Statement

The original contributions presented in the study are included in the article/supplementary material, further inquiries can be directed to the corresponding author/s.

## Author Contributions

CM, JW, and XW designed the study. XW, MY, JH, and JY conducted the study. XW, YY, and JZ collected the data. XW and YS analyzed the data and wrote the manuscript. All authors read and approved the manuscript.

## Conflict of Interest

The authors declare that the research was conducted in the absence of any commercial or financial relationships that could be construed as a potential conflict of interest.

## Publisher’s Note

All claims expressed in this article are solely those of the authors and do not necessarily represent those of their affiliated organizations, or those of the publisher, the editors and the reviewers. Any product that may be evaluated in this article, or claim that may be made by its manufacturer, is not guaranteed or endorsed by the publisher.
